# How do people conceptualize mindfulness?

**DOI:** 10.1098/rsos.211366

**Published:** 2022-03-23

**Authors:** Geoffrey Haddock, Colin M. G. Foad, Sapphira Thorne

**Affiliations:** Cardiff University, Cardiff, UK

**Keywords:** mindfulness, attributes, attitudes, values, mental representations

## Abstract

While the concept of *mindfulness* is ubiquitous, its meaning is ambiguous, with limited knowledge about how it is understood by the general public. Understanding how laypeople perceive mindfulness and mindful people is vital, as it will impact how people interpret and act upon information about mindfulness and mindfulness practices. Study 1 participants evaluated the term *mindfulness* positively, while Study 2 participants perceived a *mindful target* positively and as strongly endorsing self-transcendence values (e.g. equality). Study 3 participants learned about an unknown target who was mindful or not. The mindful target was evaluated more positively than the less-mindful target and seen as endorsing different values. Most effects in Studies 1–3 were stronger among more mindful participants. Study 4 assessed visual representations of mindful and less-mindful faces. Visual representations of a mindful face were judged by naive raters as more likeable, possessing higher self-transcendence values and performing more moral behaviours compared with a less-mindful face. The results suggest that how people interpret mindfulness has important consequences and can be used to guide how mindfulness is implemented in response to global challenges.

## 1. Introduction

Mindfulness is ubiquitous. It is incorporated in many countries' clinical, health, military and education settings, and numerous corporations use mindfulness in the workplace. In the United Kingdom (UK), an all-party government committee recommended that mindfulness provision be further increased in the health, education, justice and workplace sectors, and since 2004, mindfulness-based cognitive therapy has been recommended for clinical practice within the UK National Health Service [1]. Mindfulness practices are playing an important role in helping people deal with the coronavirus pandemic, with many countries advocating that individuals engage with self-directed mindfulness activities to help with mental health concerns brought about by the pandemic [2–4]. More generally, before the pandemic, approximately 15% of adults were thought to engage in mindfulness or meditation [5]. The continued infusion of mindfulness practices into daily activities implies that there is a widespread belief that being mindful is good. Consistent with this belief, research suggests that mindfulness has benefits for physical and mental health [6].

Yet, one glaring omission concerns how laypeople interpret and evaluate what it means to be mindful. Knowing how and what the public thinks about mindfulness is an important component in attempts to better conceptualize and operationalize the concept [7]. This is especially important as *mindfulness* has become an umbrella term, with its resultant ambiguity making it extremely difficult to characterize the concept's meaning [8]. While there have been many contemporary discussions regarding how to define mindfulness, the contributors to these discussions have largely been clinicians, practitioners and academic researchers. Prominent components of such academic and clinical definitions include (but are not limited to) increased attention awareness, increased acceptance of one's thoughts and decreased personal rumination [7–9]. Within Buddhist perspectives, concepts such as benevolence, integrity, memory and wisdom have been associated with mindfulness [8,10]. From an operational perspective, a commonly used individual difference measure of mindfulness, the five facets mindfulness questionnaire (FFMQ), has facets representing *observing* (noticing or attending to internal and external stimuli), *describing* (how people label their experiences), *acting with awareness* (attending to information in the present moment), *non-judging of inner experience* (not evaluating one's thoughts and emotions) and *non-reactivity to inner experience* (creating distance from one's thoughts and emotions) [11].

Understanding how the conceptual and operational complexity of mindfulness relates to public perceptions is particularly important, given the increasing application of mindfulness in everyday life. Individuals' willingness to potentially engage in mindfulness, their interpretation of information regarding mindfulness, and inferences they make about others who are (or are not) mindful will all depend upon their perceptions and evaluations of the concept, in the same way that variables such as individuals' expectations of health services, perceived treatment utility and trust in service providers influence uptake and adherence to in-person [12] or online health-related services [13].

With this in mind, the current research addresses the following questions: How do people perceive mindfulness and mindful individuals? What attributes and values are associated with mindful individuals? Are mindful individuals liked more than non-mindful individuals? How do people visually represent mindful and less-mindful individuals? Do these visual representations matter?

Four studies addressed these questions. Each study focused on a different level of analysis. Studies 1 and 2 adopted a bottom-up approach, where participants freely articulated their own perceptions of what it means to be mindful. Study 1 participants thought about the term mindfulness before listing and evaluating words they thought describe the concept. Study 2 participants thought about someone they considered mindful before indicating their perception of the target's attributes and values. The free-response approach used in these studies permits an assessment of participants' salient beliefs about mindfulness and mindful individuals, as well as allowing for a consideration of how these lay perceptions converge with *expert* conceptualizations (e.g. those of academic researchers, clinicians and practitioners) and common dispositional measures of mindfulness. Studies 3 and 4 adopted more of a top-down perspective in thinking about the meaning of mindfulness. Study 3 participants learned about a mindful or less-mindful target before judging and evaluating the target's attributes and values. Study 4 tested whether perceptions of mindfulness extend to visual representations that people hold of mindful and non-mindful individuals. We used the reverse correlation task [14] to assess people's visual representation of an average mindful and average non-mindful face. These faces were then shown to a separate sample, who judged the faces' attributes, values and behaviours. In all of our studies, participants completed a dispositional measure of mindfulness as well as an assessment of their own mindful experience, to assess whether these variables might impact their interpretation of what it means to be mindful and the implications of their perceptions.

All studies were approved by the Ethics Committee of the School of Psychology, Cardiff University. All analyses were carried out via SPSS (v. 26). Data are available via OSF: https://osf.io/k6w9g/?view_only=754a6c4819f949b28c76d1695c5b6342.

## 2. Study 1—how do people describe *mindfulness*?

Here, we determined the attributes people use to describe the term *mindfulness*. We assessed laypersons’ consensual responses and whether these attributes were perceived as positive or negative. Further, we considered whether individual differences in mindfulness and mindful experience might lead people to evaluate *mindfulness* in different ways. To the extent that people possess overly favourable views of attributes that are important and descriptive of their sense of self [15,16], one would expect individuals who are dispositionally more mindful to evaluate the concept more favourably than less-mindful individuals.

### 2.1. Method

#### 2.1.1. Participants^1^

One-hundred and one participants residing in the UK (62 females, 39 males; M_age_ = 36.4 years; 49% of participants reported having a UG degree or higher) were recruited through Prolific.^2^ Information about the study did not highlight that the research was related to mindfulness, in order to avoid a selection bias toward participants who engaged in mindfulness.

#### 2.1.2. Materials

Materials were presented via Qualtrics [17]. After providing consent, participants watched a 10 s video containing an image of a cartoon chicken and the sound of a cat, and participants named what they saw and heard. Anyone failing this check was screened out from the study (this was true for Studies 1–3). Next, participants were asked to consider the term mindfulness and list five words they thought suitably described the concept. After listing these words, participants rated each word's valence (re-coded for analyses: 1 = extremely negative; 7 = extremely positive). Participants then completed a secondary attribute rating task (see electronic supplementary material file for details). Next, participants completed two dispositional measures of mindfulness, the mindful attention awareness scale (MAAS) [18] and a brief version of the FFMQ [11]. The MAAS focuses on individual differences in awareness of events taking place in the present.^3^ As noted earlier, the FFMQ assesses individual differences in components of observing, describing, acting with awareness, non-judging of inner experience and non-reactivity to inner experience.^4^ Participants were asked to indicate how often, if at all, they engaged in mindfulness practice, meditation and yoga/tai chi (1 = never; 7 = always), with the items averaged together to form a measure of mindful experience. Finally, participants completed demographic information.

### 2.2. Results and discussion

#### 2.2.1. Descriptions/evaluations of mindfulness

Participants used a diverse range of terms to describe mindfulness, the most frequent of which are shown in table 1.^5^ An analysis of the consensual attributes suggests that participants describe the concept of mindfulness more in relation to being thoughtful, calm and aware and less in relation to describing or observing one's thoughts and surroundings (two facets of the FFMQ measure of mindfulness). For each participant, we computed the average of the valence ratings of their listed terms. These scores were positive (M = 5.94), with a one sample *t*-test revealing that the mean was significantly greater than the scale midpoint (4), *t*_100_ = 22.19, *p* < 0.001. That said, approximately 34% of participants provided at least one attribute they rated as neutral or negative.

**Table 1.** Responses listed 10 or more times (Studies 1–3).

Study 1 (concept)	Study 2 (target)	Study 3 (mindful James)	Study 3 (less-mindful James)
thoughtful (59)	thoughtful (46)	calm (44)	quiet (19)
aware (29)	calm (30)	thoughtful (32)	introverted (16)
calm (26)	caring (25)	careful (20)	anxious (15)
meditative (20)	kind (22)	cautious (14)	reserved (13)
peaceful (18)	careful (21)	considerate (13)	shy (13)
careful (15)	aware (15)	observant (12)	clumsy (12)
caring (15)	considerate (15)		stressed (11)
considerate (13)	cautious (11)		disorganized (10)

We also assessed whether participants' valence ratings were related to MAAS, overall FFMQ and mindful experience scores. Pearson correlations revealed that valence ratings were significantly correlated with MAAS scores, *r*_101_ = 0.21, *p* = 0.034, and mindful experience, *r*_101_ = 0.25, *p* = 0.012, with a positive but non-significant link with FFMQ scores, *r*_101_ = 0.16, *p* = 0.104.

## 3. Study 2—how do people describe a mindful individual?

In Study 2, rather than examining the *concept* of mindfulness, we focused on participants' perceptions of a *mindful exemplar*. We expected positive evaluations of mindful targets, and following from Study 1, tested whether these evaluations might be linked with participants' own level of mindfulness.

In addition, we examined whether people link being mindful with particular values. We focused on values as they are abstract ideals that serve as guiding principles in a person's life [19]. Research has demonstrated that mindful people report intrinsic values (e.g. self-acceptance) as more important than extrinsic values (e.g. wealth) [20,21]. While these findings highlight potential links between individuals' *own* mindfulness and values, research has not assessed the values people *attribute* to mindful and less-mindful individuals. This is important given the core role of perceived values in evaluating others, as greater perceived value similarity between one's self and others is associated with more positive evaluations of other individuals and groups [22].

Schwartz *et al.*'s [23] influential circumplex model differentiates among four value types. S*elf-transcendence* values reflect concern for others' welfare and include helpfulness and equality. *Self-enhancement* values reflect attention to personal status and include power and achievement. *Openness to change* values reflect pursuing personal interests in unknown directions and include self-direction and stimulation. *Conservation* values reflect preservation of the status quo and include tradition and obedience. According to Schwartz *et al.* [23], self-transcendence and self-enhancement reflect one dimension, while openness to change and conservation reflect a second dimension. We expected mindful individuals to be seen as placing most importance on self-transcendence values, an effect that might also be dependent upon participants' own level of mindfulness.

### 3.1. Method

#### 3.1.1. Participants

One-hundred and two participants residing in the UK (58 female, 43 males, 1 did not say; M_age_ = 35.9 years; 61% of participants reported having a UG degree or higher) were recruited from Prolific. Information about the study did not highlight that it was related to mindfulness.

#### 3.1.2. Materials and procedure

Materials were presented via Qualtrics. After providing consent and completing an attention check, participants thought about someone they knew whom they considered to be very mindful. They were asked to list five words to describe that person and then provide the individual's name. The attributes were evaluated for valence (re-coded for analyses: 1 = extremely negative; 7 = extremely positive). Next, participants completed the secondary attribute rating task (as in Study 1).

To measure the target's perceived values, participants completed a brief version of the Schwartz values survey (SVS) [24]. Participants indicated the extent to which their target would perceive 20 values as personally important. Five values were used for each main value type, with items (e.g. ‘How much importance would [NAMED PERSON] place on the value of EQUALITY (equal opportunity for all)?') measured on an eight-point scale (−1 = opposed to their values; 6 = very important). Finally, participants completed the MAAS, FFMQ, mindfulness experience and demographic information measures.

### 3.2. Results and discussion

#### 3.2.1. Descriptions/evaluations of the mindful target

The most frequently listed terms are shown in table 1. The most frequently listed attributes describe a known mindful target more in relation to being thoughtful, calm, caring and kind, and less in relation to describing or observing their thoughts and surroundings. Interestingly, these consensual responses are highly convergent with those provided at the concept level. For each participant, we computed a mean valence score. Like Study 1, these scores were positive (M = 6.16) and greater than the midpoint, *t*_101_ = 25.96, *p* < 0.001. That said, approximately 25% of participants provided at least one attribute they rated as neutral or negative.

We assessed whether mean valence ratings were correlated with MAAS, overall FFMQ and mindful experience. Like Study 1, valence ratings were correlated with MAAS, *r*_102_ = 0.21, *p* = 0.031, and mindful experience scores, *r*_102_ = 0.29, *p* = 0.003. The correlation between overall FFMQ scores and valence ratings was non-significant, *r*_102_ = 0.06, *p* = 0.575.

#### 3.2.2. Perceptions of the mindful target's values

We computed a mean score for each value type (*α* = 0.57–0.79, M = 0.73). First, we tested for differences in importance ratings across value types. A one-way repeated measures analysis of variance (ANOVA) revealed a significant effect, *F*_3,99_ = 100.59, *p* < 0.001. Participants perceived their target as placing more importance on self-transcendence values (M = 5.50) relative to self-enhancement (M = 3.47), openness (M = 4.06) and conservation (M = 3.76) values (all *p* < 0.001). Openness values were perceived as more important than self-enhancement values (*p* < 0.001) and marginally more important than conservation values (*p* = 0.056). Conservation values were perceived as more important than self-enhancement values (*p* = 0.050).

Finally, we considered links between participants' dispositional mindfulness, mindful experience and perceived value importance ratings. There was a tendency for dispositional mindfulness to be related to the importance of self-transcendence values, *r*_MAAS_(102) = 0.17, *p* = 0.094; *r*_FFMQ_(102) = 0.19, *p* = 0.051. Mindful experience scores tended to be linked with self-transcendence ratings, *r*_101_ = 0.17, *p* = 0.084, and openness ratings, *r*_101_ = 0.18, *p* = 0.067.

## 4. Study 3—do we prefer mindful to less-mindful people?

Study 2 offers evidence regarding how people perceive mindful individuals. Participants offered positive evaluations about a mindful exemplar and described their exemplar using terms that were highly convergent with those used to describe the concept. That said, participants in Study 2 only evaluated a known mindful target, without having them evaluate a known non-mindful target. This was done because we were concerned that any differences between evaluations of personally known and self-selected mindful and less-mindful targets might be attributable to differences independent of perceived mindfulness. For example, a participant's mindful target might have been a close friend of their age, whereas their non-mindful target might have been a distant acquaintance of a different age. To avoid this concern, Study 3 used a top-down approach that compared participants' perceptions and evaluations of an unknown target who behaved more or less mindfully. In our study, participants listened to an audio recording documenting a day in the life of a target named James. In the *mindful* condition, James engages in everyday behaviours (e.g. getting his lunch) in a mindful manner. In the *less-mindful* condition, James engages in the same behaviours, but in a less-mindful manner (designed to describe an average individual). After learning about James, participants listed and evaluated attributes they felt described him and indicated their perceptions of his values.

We expected mindful James to be evaluated more favourably and judged as holding different values than less-mindful James and tested whether these judgements would be influenced by participants' own mindful tendencies.

### 4.1. Method

#### 4.1.1. Participants

One-hundred and ninety-six participants residing in the UK (123 female, 72 male, 1 did not say; M_age_ = 36.3 years; 52% of participants reported having a UG degree or higher) were recruited from Prolific. Information about the study did not highlight that the research was related to mindfulness.

#### 4.1.2. Materials

Materials were presented via Qualtrics. After providing consent, participants watched the attention check video before being randomly assigned to listen to one of two 3 min audio recordings. In both versions, James performed routine tasks (e.g. driving to work, getting his lunch). The sole difference was whether James' behaviour was or was not mindful. For example, in the mindful condition, James’ lunchtime was described as follows:

At lunchtime, James goes for his usual stroll from the office to the nearby sandwich store. It is about a 5 or 6 min walk, through a small park. As he is walking, he looks around and notices many things that interest him about his surroundings, including three different birds singing in one tree and the range of different plants that have just been put in the flower-beds by the local council.

In the less-mindful condition, James' lunchtime was described as follows:

At lunchtime, James goes for his usual stroll from the office to the nearby sandwich store. It is about a 5 or 6 min walk, through a small park. As he is walking, he puts his headphones on to listen to one of his favourite songs.

Participants then were asked to list and evaluate five words they felt best described James (re-coded for analyses: 1 = extremely negative; 7 = extremely positive). As in Studies 1 and 2, participants completed the secondary attribute task. Participants then completed an SVS measure of their perceptions of James' values. They then completed the MAAS, FFMQ and mindful experience measures before completing demographic information.

### 4.2. Results and discussion

#### 4.2.1. Descriptions/evaluations of James

Mindful James was described using attributes comparable to those elicited in Studies 1 and 2; less-mindful James was seen as quiet, introverted, anxious and reserved (table 1). A *t*-test revealed that mindful James (M = 5.99) was evaluated more positively than less-mindful James (M = 4.07), *t*_152.6_ = 11.35, *p* < 0.001. While mindful James (M = 5.99) was evaluated more positively than the scale midpoint, *t*_93_ = 25.10, *p* < 0.001, less-mindful James (M = 4.07) was not, *t*_101_ = 0.46, *p* = 0.647. In the mindful condition, approximately 37% of participants provided at least one attribute they rated as neutral or negative; the comparable figure in the less-mindful condition was approximately 87%.

Next, we assessed whether valence ratings were correlated with MAAS, FFMQ and mindful experience. For mindful James, scores on both the MAAS, *r*_94_ = 0.26, *p* = 0.012, and FFMQ, *r*_94_ = 0.36, *p* < 0.001, were correlated with evaluations, with more mindful participants being more positive in their assessment. Mindful experience was not correlated with evaluations of mindful James, *r*_94_ = −0.09, *p* = 0.407. None of the variables were linked with evaluations of less-mindful James (all *p* > 0.440).

#### 4.2.2. Perceptions of James' values

We computed scores for each value type (*α* =0.74–0.89; M = 0.83) and compared whether mindful James and less-mindful James were perceived as holding different values. A 2 (condition) × 4 (value type) mixed-ANOVA revealed a main effect of condition, *F*_1,194_ = 15.43, *p* < 0.001. Overall, higher value importance ratings were ascribed to mindful James than less-mindful James. There was also a main effect of value type, *F*_3,582_ = 230.90, *p* < 0.001. All comparisons across value types were significant (*p* < 0.001). However, these main effects were qualified by a significant interaction, *F*_3,582_ = 12.04, *p* < 0.001. Regarding the self-transcendence and self-enhancement dimension, mindful James was perceived as valuing self-transcendence more than less-mindful James (M_mind_ = 5.25; M_less-mind_ = 4.17; *F*_1,194_ = 39.60, *p* < 0.001), with no difference for self-enhancement values (M_mind_ = 3.54; M_less-mind_ = 3.37; *F*_1,194_ = 0.92, *p* = 0.339). Regarding the openness and conservation dimension, mindful James was perceived as valuing openness more than less-mindful James (M_mind_ = 2.96; M_less-mind_ = 2.57; *F*_1,194_ = 4.18, *p* = 0.042); mindful James was also perceived as valuing conservation values more than less-mindful James (M_mind_ = 4.54; M_less-mind_ = 3.92; *F*_1,194_ = 15.83, *p* < 0.001). This pattern suggests that participants perceived mindful James to attach greater importance to more selfless values.

We also assessed whether MAAS, FFMQ and mindful experience were linked with perceived value importance ratings. For mindful James, high MAAS participants perceived him as placing more importance on self-transcendence, *r*_94_ = 0.20, *p* = 0.053, self-enhancement, *r*_94_ = 0.23, *p* = 0.025, and openness values, *r*_94_ = 0.23, *p* = 0.025. Higher FFMQ scores were associated with perceiving mindful James as placing higher importance on self-transcendence values, *r*_94_ = 0.23, *p* = 0.029. Participants with more mindful experience perceived mindful James as valuing conservation values as more important, *r*_94_ = 0.24, *p* = 0.019. There was one marginally significant effect for less-mindful James—higher FFMQ scores tended to be linked with lower self-enhancement ratings, *r*_102_ = −0.18, *p* = 0.066.

## 5. Study 4—what does a mindful person look like?

Studies 1–3 offer novel conceptual evidence regarding how people think about mindfulness and mindful individuals. Study 4 extends these results by addressing participants' *visual representation* of a mindful individual—that is, perceptions of what a mindful person *looks like*. We tested whether people have different mental images of mindful and non-mindful individuals and whether other people, if shown an average mindful face and average non-mindful face, would associate these faces with different attributes, values and behaviours. Such differences would speak to basic, fundamental conceptualizations of mindfulness, consensual perceptions of mindfulness and implications of simply being *perceived* as mindful.

We addressed these questions using the reverse correlation task [14]. The task involves one group of participants generating their own visual representation of group members, in our case mindful and non-mindful individuals. These representations are averaged across participants, with one classification image representing the average facial image of a category member and a second classification image representing the average facial image of a category non-member. These average images are then evaluated by another sample of participants, unaware of how the faces were generated.

This task has been used to study representations of various categories [25,26]. After deriving an image of an average welfare recipient, Brown-Iannuzzi *et al*. [25] found that participants judged this face as more African American, lazy and incompetent than a non-welfare recipient face. Brown-Iannuzzi *et al*. [26] found that an average atheist face was judged as less competent, likeable and happy than an average theist face. The average atheist face was also judged as more likely than the average theist face to commit immoral acts.

In phase one of our study, one sample of participants generated a facial image of a mindful and non-mindful person. In phase two, a separate sample rated these images on a series of attributes, measures of likeability, warmth and competence, as well as the Big Five. Participants also indicated their perceptions of the values of the mindful and non-mindful faces and made decisions about the likelihood of these two people committing moral and immoral acts.

We expected the mindful face to be judged more favourably than the non-mindful face, as attaching greater importance to self-transcendence values and less importance to self-enhancement values, and being more likely to commit moral acts. We also considered whether any such effects might depend on participants' own level of mindfulness.

### 5.1. Method

#### 5.1.1. Image generation phase

*Participants.* One-hundred and thirteen participants (104 females, 9 males; M_age_ = 19.5 years) were recruited via a British university. As in Studies 1–3, information about the study did not mention mindfulness.

*Materials and procedure.* The task was presented on E-Prime 3.0. Participants completed 400 trials. On each trial participants were shown two 400 by 400 px facial images; one image was a base face with a certain noise pattern superimposed, the second had the reverse noise pattern superimposed onto the same base face. The random noise was generated and added using the rcicr package in *R* [27]. The images were created from a single morphed base face that combined a facial image of a Black man, a White man, a Black woman and a White woman [25,26]. For each image pair, participants selected which image looked the most mindful. They were given 10 s to respond before moving to the next trial. A trial ended after 10 s or a participant's response. Each trial used a different pair of images, and pairs of stimuli were presented in a random order. Participants could take a break after every 100 trials.

Finally, participants completed a brief version of the FFMQ, items regarding mindfulness experience, religion and demographic information.

#### 5.1.2. Image processing

**Figure 1.**
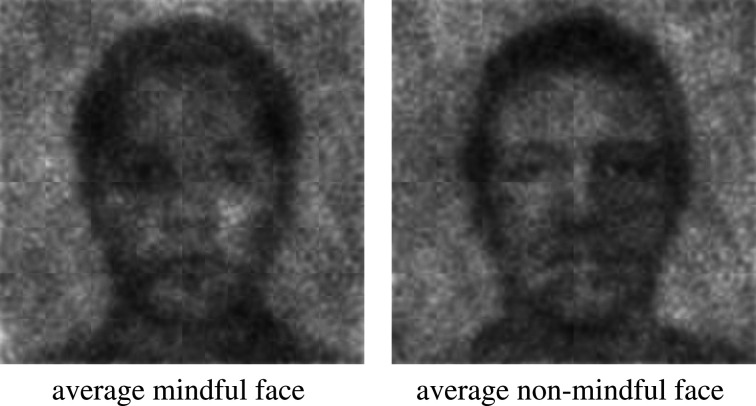
Average classification images.

We computed average mindful and non-mindful faces using the rcicr package in *R* [27]. The mindful face was created by superimposing the composite base face image with noise patterns that were averaged across the images selected by individual participants. The non-mindful face was computed in a similar way but used the noise patterns averaged across images *not* selected by individual participants. The average mindful and non-mindful images are presented in figure 1.

#### 5.1.3. Image rating phase

*Participants.* Two-hundred and eighty individuals residing in the UK were recruited from Prolific. Information about the study did not highlight that the research was related to mindfulness. Seventeen individuals were excluded for failing an attention check; the final sample was 263 (178 female, 83 male, 2 other; M_age_ = 34.3 years; 36% non-religious, 31% Christian, 11% atheist; 9% agnostic; 10% all other categories).

*Materials and procedure.* Materials were presented via Qualtrics. After providing consent, participants were presented with 10 different activities that people like to engage in, accompanied with the question ‘Which of these activities do you engage in to relax (click on all that apply)?' However, the instructions preceding this section told participants that in order to progress to the study they needed to select ‘none of the above'. Anyone failing this check was given another opportunity to read the instructions and try again. A second failure led to being screened out from the study.

Next, participants were told that they would be making judgements about visually distorted images. The images were not described in any way—thus participants reported their perceptions *without* knowing that the two faces represented average mindful and non-mindful faces. There were four parts to the rating phase. First, participants were informed that they would see blurred image pairs and asked which of the pair most fit different attributes: mindful, likeable, feminine, masculine and attractive (e.g. ‘Which image looks most like a mindful person?'). The mindful attribute acted as a manipulation check, all other attributes assessed more global evaluations of the two facial images. These attributes were presented in a random order, each on a separate screen. Participants made decisions for the paired mindful and non-mindful images, as well as a pair of filler images. The filler pair was included to reduce the salience of the two critical mindful images [26]. Participants completed all attributes for the filler faces as well as the mindful and non-mindful face. We counterbalanced the side that the mindful face was presented across participants.

Second, participants rated the mindful and non-mindful faces on the following attributes (based on Study 3): calm, thoughtful, rational, quiet, stressed and normal. Participants also rated both images on warmth, competence, likeability, meditative and the Big 5 (1 = not at all; 6 = extremely) [28]. Each attribute for each image was presented individually, in a random order.

Third, participants rated their perceptions of the values held by the mindful face and the non-mindful face. Participants indicated the extent to which the person would perceive four different values as important, compared with the average person in the UK. We used two values assessing self-transcendence (universalism, benevolence) and two other values assessing self-enhancement (power, achievement). Participants provided their perceptions of each value using a sliding scale (0 = less important to them than to the average person living in the UK; 100 = more important to them than to the average person living in the UK). Preceding the values measure, participants were presented with the image of the face they were to make judgements about. Participants made judgements about both the mindful and non-mindful images, and image order was counterbalanced across participants.

Fourth, participants judged which of the two images was more likely to perform various behaviours. We included four moral (e.g. volunteers 3 hr a week at a homeless shelter) and four immoral (e.g. refused to help a homeless person) behaviours adapted from Brown-Iannuzzi *et al*. [26]. For each scenario, participants were shown the mindful and the non-mindful faces and selected which one was more likely to have performed this behaviour. We counterbalanced the side that the mindful face was presented across participants.

Finally, participants completed a brief version of the FFMQ and questions regarding their mindfulness experience, religion and demographic information.

### 5.2. Results and discussion

#### 5.2.1. Global perceptions

As expected, participants considered the mindful image as more mindful than the non-mindful image, $\chi_{1}{}^{2}=51.50$, *p* < 0.001. The mindful face was also considered as more attractive, $\chi_{1}{}^{2}=30.56$, *p* < 0.001, and likeable, $\chi_{1}{}^{2}=38.57$, *p* < 0.001, the latter consistent with the positive evaluations of mindful James in Study 3. The mindful face was also considered more feminine, $\chi_{1}{}^{2}=142.64$, *p* < 0.001, and less masculine $\chi_{1}{}^{2}=148.41$, *p* < 0.001.

#### 5.2.2. Ratings of attributes

We first tested whether ratings of mindful and non-mindful attributes differed for the mindful and non-mindful faces. The mindful face was rated as more calm, thoughtful and rational than the non-mindful face (all *p* < 0.001; top section of table 2). The non-mindful face was perceived as more stressed relative to the mindful face (*p* < 0.001). Interestingly, the mindful face was rated significantly more quiet and normal relative to the non-mindful face (both *p* < 0.001).

**Table 2.** Ratings of mindful and non-mindful faces on attributes (Study 4).

attribute	mindful face		non-mindful face		*t*	*p*
	M	95% CI	M	95% CI		
calm	4.06	[3.92, 4.19]	3.22	[3.06, 3.39]	8.40	<0.001
thoughtful	3.82	[3.70, 3.93]	2.67	[2.53, 2.81]	14.19	<0.001
rational	3.72	[3.60, 3.84]	2.92	[2.80, 3.05]	9.63	<0.001
quiet	4.47	[4.33, 4.61]	3.68	[3.51, 3.85]	7.45	<0.001
stressed	2.84	[2.68, 3.00]	3.38	[3.22, 3.54]	−5.43	<0.001
normal	4.32	[4.18, 4.45]	3.81	[3.65, 3.97]	6.76	<0.001
						
likeable	3.70	[3.56, 3.83]	2.42	[2.29, 2.55]	14.64	<0.001
competent	3.65	[3.53, 3.77]	3.21	[3.08, 3.33]	5.42	<0.001
warm	3.30	[3.14, 3.45]	1.98	[1.87, 2.10]	13.44	<0.001
extraverted	2.59	[2.46, 2.72]	2.68	[2.53, 2.83]	−0.96	0.336
conscientious	3.63	[3.51, 3.75]	2.65	[2.52, 2.79]	11.17	<0.001
openness	3.24	[3.10, 3.37]	2.39	[2.25, 2.52]	9.98	<0.001
agreeable	3.71	[3.58, 3.85]	2.38	[2.26, 2.50]	15.33	<0.001
neurotic	2.77	[2.62, 2.92]	3.43	[3.27, 3.59]	−6.49	<0.001
meditative	3.21	[3.08, 3.35]	2.30	[2.16, 2.43]	10.18	<0.001

Next, we tested whether perceptions of likeability, competence, warmth and the Big 5 differed across the mindful and non-mindful faces. The mindful face was rated as more likeable, competent and warm than the non-mindful face (all *p* < 0.001; bottom section of table 2). Further, the mindful face was perceived as more conscientious, open, agreeable and meditative, while the non-mindful face was perceived as more neurotic compared with the mindful image (all *p* < 0.001). When controlling for multiple comparisons, FFMQ scores and mindful experience were not significantly correlated with image ratings and ratings of attributes (all *p* > 0.024).

#### 5.2.3. Perceptions of values

We computed separate indices reflecting self-transcendence and self-enhancement values and assessed differences in the perceived importance of these values for the mindful and non-mindful faces. A 2 (mindful versus non-mindful face) × 2 (self-transcendent versus self-enhancement values) repeated measures ANOVA revealed that participants assigned higher value ratings to the mindful face than the non-mindful face, *F*_1,262_ = 79.12, *p* < 0.001. There was also a main effect of value type; participants gave higher ratings for self-enhancement than self-transcendence values, *F*_1,262_ = 33.10, *p* < 0.001. However, as expected, the main effects were qualified by a significant interaction, *F*_1,262_ = 255.28, *p* < 0.001. The mindful face (M = 58.60) was perceived as placing more importance on self-transcendence values compared with the non-mindful face (M = 33.14), *t*_262_ = 16.27, *p* < 0.001. The opposite pattern was found for self-enhancement values, with the non-mindful face (M = 58.95) being perceived as placing more importance on these values compared with the mindful face (M = 45.61), *t*_262_ = −11.16, *p* < 0.001. Participants' ratings of values were unrelated to their FFMQ scores or mindful experience (*p*s > 0.293).

#### 5.2.4. Moral behaviours

Similar to [26], participants were assigned a score of 1 when they selected the mindful face as more likely to have performed the behaviour and a score of 0 when they selected the non-mindful face as more likely to have performed the behaviour. A paired sample *t*-test found that participants were more likely to select the mindful face when the behaviour was moral (M = 0.79) than immoral (M = 0.18), *t*_262_ = 19.40, *p* < 0.001). Participants' selections were unrelated to their scores on the FFMQ and mindful experience (*p*s > 0.289).

## 6. General discussion

In the light of discussions regarding ambiguity in conceptualizations of mindfulness, the current research considered how laypeople construe mindfulness and mindful people. Because mindfulness programmes are increasingly used across clinical, health, education and corporate sectors, it is important to understand how people interpret what it means to be mindful. However, such representations are not understood and will have a direct influence on individuals' potential willingness to engage in mindfulness, their interpretation of information and evidence regarding mindfulness and inferences they make about others who are (or are not) mindful (see [12,13], for relevant evidence outside the realm of mindfulness).

Across our studies, several novel and conceptually important findings were observed. First, there was consistency in the content and valence of self-generated attributes associated with mindfulness (Study 1) and mindful people (Study 2). Mindfulness, and mindful individuals, were perceived positively by participants, though many respondents provided one or more attributes they believed were neutral or negative. Further, participants' evaluations of what it means to be mindful were positively linked with their own self-reported mindfulness, as assessed by the MAAS (and to a lesser extent the FFMQ). This was found for the concept, a self-generated mindful target and an unknown mindful target. This is consistent with research demonstrating that people have favourable views of concepts that are central to their own self-schema [15,16]. This may also reflect mindful individuals being likely to see the benefits of mindfulness for themselves and attributing these benefits to others. Interestingly, this pattern of results was not found in Study 4, which involved a less-reflective process.

The results of Studies 1 and 2 offer evidence that people's freely elicited responses of what it means to be mindful are most attuned to the awareness, calm and thoughtful component of mindfulness, as well as attributes linked to loving-kindness, a form of meditation linked with cultivating positive emotions [29]. Thus, overall there is reasonable overlap between lay and clinical conceptualizations of mindfulness, and mapping such similarities and differences lays foundations for future work, which would also benefit from a more detailed consideration of how lay conceptualizations map onto broader Eastern conceptualizations of mindfulness. At the same time, understanding how public perceptions compare with expert views has important implications in relation to how mindfulness is measured and implemented. Regarding measurement, our findings suggest that public representations are not necessarily linked with all elements of mindfulness that are captured in many prominent dispositional measures (e.g. the observing and describing facets of the FFMQ). Regarding implementation, fully understanding public perceptions is especially important, as many governments and organizations are advocating mindfulness practices to help manage public health in response to COVID-19 [2–4]. By incorporating into their message the public's understanding of what mindfulness is (and is not), it is likely to impact individuals' receptivity to mindful practices.

To our knowledge, Study 4 represents the first research assessing individuals' visual representations of mindful people. Spontaneously generated representations from one sample influenced judgements made by other, naive participants, with the average mindful face evaluated more positively on core attributes, values and behaviours. Thus, simply *seeing* a consensual representation of a mindful face has substantial implications, even when participants are unaware of the source of the representation and there was no information linking the visual representation with the concept. This implies that people seem to have well-developed representations of what it means to be mindful, that these representations appear to be shared across individuals and have fundamental implications for interpersonal judgements. As noted by Brown-Iannuzzi *et al*. [26], visual representations can serve as subtle mechanisms that link categories and evaluations. Our results extend this reasoning to mindfulness.

Another important finding concerns link between mindfulness and values. In Studies 2 and 3, mindful targets were perceived as placing particular importance on self-transcendence values, values reflecting concern for others. In Study 4, the average mindful face was judged as possessing self-transcendent values to a greater extent, while the non-mindful face was judged as possessing self-enhancement values to a greater extent. Thus, simply being seen as mindful leads others to infer that one attaches greater importance to ideals such as helpfulness and equality, whereas being perceived as less mindful leads others to infer that one attaches greater importance to ideals such as authority and ambition. Further, these inferences have important consequences, as perceptions of others' values influence how they are judged, with larger perceived value differences between ourselves and others linked with outcomes such as prejudice [22].

Our studies, with the exception of image generators in Study 4, used UK-based public samples, because we sought to obtain a broader view of individuals' perceptions and evaluations of mindfulness. This is particularly important given the overarching aims of the research — to understand how the public perceives mindfulness and mindful individuals, the visual representations associated with mindful and less-mindful individuals, and how these perceptions and representations have meaningful implications. That said, while the use of public non-student samples is a strength of the work, future research could address potential cross-cultural differences.

Overall, this research represents a new approach to understanding what it means to be mindful. The results suggest that laypersons have consensual views on what it means to be mindful. Further, mindful people are preferred to less-mindful people, perceived as more likely to possess self-transcendence values and more likely to engage in moral behaviours. These effects are found when participants reflect upon individual targets and when making naive judgements simply based on faces that are mindful or non-mindful.

## Data Availability

Data are available via OSF, at https://osf.io/k6w9g/?view_onl=754a6c4819f949b28c76d1695c5b6342.

## Footnotes

1 Across our studies, 81 individuals who consented to participate failed an attention check and could not proceed. Fifty-two individuals who consented to participate dropped out before completion.

2 Individuals could participate in only one of our studies.

3 Because some of our participants did not have a driver's licence, we excluded from analyses the MAAS item referring to driving behaviour.

4 Four MAAS items were among the FFMQ items that we selected.

5 Please see the elctronic supplementary material for how we coded words similar in meaning.
